# Readability of U.S. food handler training materials: a natural language processing analysis of worker study guides and the federal Food Code

**DOI:** 10.3389/fpubh.2026.1911335

**Published:** 2026-08-27

**Authors:** Morris Brako, Anirudh R. Naig

**Affiliations:** Department of Apparel, Events, and Hospitality Management, Iowa State University, Ames, IA, United States

**Keywords:** Americans with Disabilities act, cognitive load theory, food handler certification, food safety training, health literacy, plain language, readability

## Abstract

**Introduction:**

In many U.S. jurisdictions a food handler card requires studying a training document and passing a written examination; one state legally requires that the document be furnished to every applicant. Whether these documents can be read by the workers required to use them is unknown. The common assumption that certification is required in all fifty states is also inaccurate: among the jurisdictions whose rules we verified, some mandate training but delegate its content to private vendors, so no worker-facing manual is publicly available.

**Methods:**

A natural language processing pipeline (pdfplumber, spaCy, textstat) was applied to 20 documents retrieved under a documented protocol: 14 worker-facing study guides across 11 jurisdictions in eight states, the 2022 FDA Food Code, and five Spanish-language counterparts, excluded from analysis. These jurisdictions contain roughly 26.7 million residents, about 8 percent of the U.S. population. The document was the unit of analysis. All 417 sentences of 80 tokens or more were audited, and results are reported under three extraction rule sets.

**Results:**

One document stood apart. Washington’s Food Worker Manual met the recommended sixth-grade Flesch–Kincaid target under all three extraction rule sets, at a mean sentence length of 10.6 words, while addressing the same ten content domains as the other guides. The remaining 13 of 14 guides exceeded that target under every rule set (guide mean 10.33, 95% CI 9.15–11.11), and at most one met the Gunning Fog target of 8 (mean 13.08, 95% CI 11.85–13.91). The Food Code was more demanding than every guide (Flesch–Kincaid 15.30; Gunning Fog 19.02), with mean sentence lengths of 38.87 words against 25.00.

**Discussion:**

The Washington result is consistent with the cognitive load account that motivated the measures and indicates that lower reading levels are attainable in this domain, giving agencies a working model rather than an exhortation. Where materials are delegated to private vendors, their accessibility cannot be audited. Two states have adopted alternatives to the written examination under the Americans with Disabilities Act, differing in whether they preserve the holder’s occupational range. Readability formulas measure textual difficulty, not comprehension; direct testing with workers is the necessary next step.

## 1 Introduction

### 1.1 Foodservice employment and the literacy demands of certification

The foodservice industry is often described as an accessible entry point into paid work, particularly for populations that face barriers in other sectors. Its concrete task sequences, predictable routines, and clearly defined roles align with the supported employment model that has guided inclusive workforce policy for several decades (1–3). Access to that entry point is uneven: the Bureau of Labor Statistics reports an employment-to-population ratio of 22.5% for people with a disability against 65.8% for those without (4), a gap that persists across industries including foodservice.

Entry to the sector is also mediated by a documentary requirement. A substantial share of entry-level foodservice employees have limited formal education, and food safety training has been shown to disadvantage low-literacy workers specifically (5). Where a jurisdiction requires a food handler card, the pathway typically runs through a worker-facing guide and a written knowledge examination (6). For a confident reader this is an administrative hurdle. Where reading fluency is limited, the difficulty of the study material itself can become the binding constraint on obtaining the credential, and therefore on employment.

Prior readability research in food safety has concentrated on consumer-facing pamphlets rather than the study guides workers actually use to obtain certification (7). This study addresses that gap.

### 1.2 Cognitive load theory and instructional text

Cognitive Load Theory (CLT) offers a well-validated account of why document design matters for learning (8, 9). Working memory is strictly capacity-limited (10, 11), and CLT separates the load a learner experiences into intrinsic load, arising from the inherent difficulty of the content; extraneous load, generated by how the material is presented; and germane load, the effort that builds durable knowledge (12). Extraneous load contributes nothing to learning and competes directly with the capacity available for the other two.

Food safety instruction carries substantial intrinsic load. A worker must hold simultaneously a set of numeric thresholds, a set of conditional rules whose antecedents are themselves technical, and a causal model connecting pathogens to practices. That load is irreducible; it is what the training exists to convey. What is not irreducible is the extraneous load added on top of it by long sentences, dense vocabulary, and continuous exposition where enumerated steps would serve.

This distinction does more than motivate the study. Because CLT locates the remediable problem in extraneous rather than intrinsic load, it yields a testable prediction: the same content should be deliverable at substantially lower processing cost without simplifying the content itself. That prediction determines which textual properties are worth measuring: a matter taken up in Section 2.6.1, where each metric is selected for the load channel it indexes; and it is tested against the corpus in Section 4.2.

### 1.3 Plain language standards for public-facing materials

Universal Design for Learning calls for multiple means of representation so that learners with different profiles can reach the same content (13, 14). Health communication guidance points the same direction. The Plain Writing Act of 2010 requires plain language in federal public-facing documents (15), the CDC recommends a sixth-grade reading level or lower for general audiences (16), and the Agency for Healthcare Research and Quality recommends a fourth-to-sixth grade level for consumer health information (17). Evidence supports these targets: materials written at lower reading levels improve comprehension and recall among readers with limited health literacy (18). Whether the documents that govern food handler certification meet these standards has not been established empirically.

### 1.4 What jurisdictions actually publish

A claim repeated in secondary sources and in the training industry’s own materials holds that food handler or food manager certification is required in all fifty states. Verification against primary administrative sources shows this to be inaccurate, and the inaccuracy conceals something consequential for any attempt to study these materials.

U.S. jurisdictions fall into three regimes. Some publish a free, public, worker-facing manual. Some mandate worker training but delegate its content to private accredited vendors, whose materials are copyrighted and priced. Some require only a certified food protection manager and issue no worker-level material at all. Only the first group produces documents that any researcher, advocate or worker can obtain and examine without cost or permission. Vendor materials may sometimes be purchased or released on request, but their accessibility cannot be assessed systematically or independently, and a worker cannot inspect them before deciding whether the training is usable.

This has a direct methodological consequence, developed in Section 2.2: the population of publicly examinable worker-facing training documents is far smaller than the population of jurisdictions with training mandates, and any readability finding necessarily describes the transparent tier. It also has a substantive one, developed in Section 4.3.4: where content is delegated, the accessibility of a legal requirement becomes a matter of private discretion and cannot be audited by anyone.

### 1.5 Objectives

This study had two aims:

The first was to quantify the linguistic complexity of publicly available worker-facing food handler training documents, alongside the 2022 FDA Food Code as the regulatory text they operationalize, measured against established plain-language targets, analyzed at the document level, and interpreted through cognitive load theory.

The second was to characterize the jurisdictional supply of such documents, which states publish worker-facing material, which delegate it, and which require no worker-level training; so that the corpus rests on an explicit sampling frame rather than on convenience.

A third contribution emerged from the second. In locating the administrative rules that determine each jurisdiction’s regime, we identified provisions in two state food codes that establish non-written-examination pathways to a food worker card, grounded explicitly in the Americans with Disabilities Act. To our knowledge these have not been described in the published literature. They are reported in Section 4.3.3 as a regulatory audit rather than as a claim about any population of workers.

## 2 Materials and methods

### 2.1 Design

This is a cross-sectional textual analysis of publicly available food handler training materials issued by U.S. public health authorities, together with the federal regulation those materials operationalize. The design is descriptive and comparative at the level of the document: each document contributes one observation to each analysis, and all inferential claims concern the population of publicly available worker-facing training documents rather than the sentences within any particular document.

Two properties of the object of study shaped the design. First, the documents are not a sample drawn from a well-defined list, because no such list exists; the sampling frame had to be constructed, and Section 2.2 describes how. Second, the materials are legally consequential, in at least two jurisdictions their distribution to workers is compelled by administrative rule, so the analysis treats them as regulatory instruments whose comprehensibility is a matter of statutory implementation, not merely as texts.

Readability formulas are used throughout as measures of *textual difficulty*, operationalized as surface features of words and sentences. Their strengths and limits as computational instruments are well characterized (19), including in regulatory and legal text specifically (20). They are not measures of comprehension. No participant read any document in this study, and no claim is made about what any worker did or did not understand. Section 2.6 states the metric selection rationale and Section 5 the limits this places on inference.

### 2.2 Sampling frame: a taxonomy of jurisdictional training regimes

#### 2.2.1 How states provide training materials

Secondary sources and commercial training providers commonly state that food handler or food manager certification is required in all fifty states (6). Verification against primary administrative sources shows that this characterization is inaccurate, and its inaccuracy is structural rather than incidental: U.S. jurisdictions differ not only in whether they mandate worker-level training but in whether any worker-facing material exists to examine. A corpus of training documents therefore cannot be assembled without first establishing which jurisdictions produce such documents at all.

We classify jurisdictions into three regimes:

Publisher: The jurisdiction issues a free, public, worker-facing manual or study guide. These are the only jurisdictions whose training content can be independently examined by a researcher, a worker, or an advocate.

Delegator: The jurisdiction mandates worker training but delegates content to private accredited vendors (for example ServSafe, StateFoodSafety, Learn2Serve). Materials are copyrighted, priced, and distributed under licence. Their readability cannot be assessed by anyone outside the vendor.

Manager_only: The jurisdiction requires only a Certified Food Protection Manager and imposes no worker-level training mandate, so no worker-facing instructional document is issued.

The taxonomy is not merely an inclusion device. It identifies a structural asymmetry in public accountability: in DELEGATOR jurisdictions the accessibility of legally mandated training material is unauditable in principle, and the absence of evidence about those materials is itself a finding, reported in Section 4.

#### 2.2.2 Classification procedure and current coverage

Regime classification for a jurisdiction required locating and reading (a) the state food code or equivalent administrative rule, (b) any food worker card or permit rule, and (c) the issuing agency’s public materials page. Classification was recorded in a structured census (census.json) with the governing citation, manual URLs where present, available languages, and any accommodation provision identified. Search patterns are documented in the deposited materials; ADA accommodation provisions in particular cannot be located by a single query string, since jurisdictions use unrelated terminology for the same mechanism (Washington: “limited duty card”; Alaska: “waive the examination requirement”), so each jurisdiction’s rules were read individually.

##### 2.2.2.1 Coverage is partial and is reported as such

At the time of analysis, regime classification had been verified against primary sources for eight of fifty-one jurisdictions (the fifty states and the District of Columbia): Alaska, Idaho, Illinois, New Mexico, Oregon, Texas, Utah, and Washington. Document retrieval was additionally pursued in jurisdictions identified as publishing worker-facing materials at county level, including counties in California, Nevada, Oklahoma, and Virginia. The census remains open for the remaining jurisdictions.

We therefore make no claim that the corpus constitutes a census, or a near-census, of publisher jurisdictions. The defensible statement is narrower and is the one we make: the corpus comprises the worker-facing training documents that a documented systematic search identified as publicly retrievable, and inference is confined to that population, publicly available worker-facing guides, rather than to all training material delivered to U.S. food workers, most of which is privately held and cannot be examined.

#### 2.2.3 Inclusion and exclusion criteria

A document was included if it satisfied all of the following, assessed before any readability metric was computed:

1. It is instructional material addressed to food workers, as distinct from regulatory text addressed to operators or inspectors, administrative forms, application instructions, or promotional material. 2. It is issued or formally adopted by a governmental public health authority, or by a body operating under that authority’s approval, and is offered as preparation for a jurisdiction’s food handler requirement. 3. It is publicly retrievable without payment, registration, or licence. 4. It is a complete document rather than an excerpt or summary page.

The 2022 FDA Food Code (21) was included separately as the federal regulatory reference that the worker-facing tier operationalizes. It is not a worker-facing guide and is never pooled with them.

Two consequences of criterion 2 should be stated explicitly, since the corpus contains documents that sit at its edge. The Oregon State University extension guide is produced by a land-grant university rather than a health department, and the Oregon eFoodcard study guide is produced by a state-approved provider; both are included because they are offered to Oregon workers as preparation for the state requirement and are freely retrievable, which is the property the study is about. Descriptions of the corpus in Results are worded to reflect this rather than attributing every document to a health department.

Criterion 1 is defined independently of whether a jurisdiction mandates worker certification. Idaho publishes a free worker manual without mandating worker-level certification, and is included: the study concerns the readability of material offered to workers, not the legal force behind it. Idaho is analytically informative precisely because it inverts the delegator pattern, and because the state’s own guidance states that the examination’s answers are contained in the manual, which makes the manual’s readability directly determinative of examination outcomes.

A document was excluded if it was an inspector-facing or operator-facing document, an administrative page retrieved in error, a duplicate of a document already held (detected by SHA-256 of file bytes), or a Spanish-language document where English-calibrated formulas would be applied, see Section 2.7.

### 2.3 Retrieval protocol

Retrieval was performed by an instrumented harness (01_retrieve.py) against a candidate URL list (candidates.json) holding, for each jurisdiction, the primary agency URL and one or more documented fallbacks such as CDN or county mirrors. For every attempt, whether successful or not, the harness recorded the jurisdiction, requested URL, UTC timestamp, attempt number, HTTP status code, final URL after redirects, declared content type, payload size in bytes, SHA-256 digest of the retrieved bytes, presence of a PDF file header, presence of an end-of-file marker, page count where parseable, and a disposition. The complete log (retrieval_log.csv) is deposited and constitutes the reproducibility record for corpus assembly.

Integrity checking was applied to every retrieved object rather than assumed from the file extension. This is consequential: one candidate object served with a .pdf extension was found on inspection to be a JPEG image (magic bytes FF D8 FF E0, Content-Type: image/jpeg, HTTP 200), and was excluded rather than being recorded as a corrupt PDF. Two further objects retrieved from state agencies parsed cleanly as complete, valid PDFs but proved on reading to be administrative pages rather than worker manuals; these were retrieval-targeting errors on our part and were excluded as out-of-scope documents, not as failures of the serving agency.

We record these outcomes as corpus-assembly documentation and draw no inference from them. The number of retrieval attempts is far too small to support any claim about the reliability of government document delivery, and the outcomes, correctly diagnosed, describe our targeting rather than agencies’ performance.

One observation is recorded without inferential weight, because it bears on reproducibility: several agency URLs that served these documents earlier in the study period returned HTTP 404 on re-retrieval, while CDN and county mirrors continued to serve byte-identical files. Readers attempting to reproduce corpus assembly should expect to use the deposited hashes rather than the original URLs.

### 2.4 Text extraction

Text was extracted from PDF sources with pdfplumber. Pages were retained only if they contained at least 25 words and had a digit-to-character ratio below 0.30. The word floor removes cover pages, section dividers, and pages consisting of a single image with a caption; the digit ceiling removes temperature charts, indices, and tabular reference pages whose extracted token stream is not prose and whose inclusion would corrupt sentence segmentation. A ceiling of 700 pages per document was configured; no document in the corpus reached it, so the ceiling was not binding on any result.

The page ceiling was set high deliberately. A ceiling low enough to bind would truncate long documents from the front, and for the 2022 FDA Food Code, which runs to 668 pages, front truncation would restrict analysis to its definitional and preambular sections, which are its densest legal prose. Truncation of that kind is not neutral with respect to the quantities measured here, so every document is analysed in full.

Page retention is reported per document in the deposited validation report. Retention among PDF-sourced documents ranged from 80.8 to 100%, with a median near 94%; the Food Code retained 663 of 668 pages.

One document was not obtained as a PDF, and this asymmetry is material enough to state prominently. The Washington State Food Worker Manual circulates as a county-mirrored PDF that is a scanned image with no text layer on any of its 30 pages. That artifact is machine-unreadable, and analyzing it was not possible. That artifact also fails the accessibility requirements that apply to government digital content: an image-only PDF cannot be read by a screen reader, searched, or machine-translated, which is the class of barrier Section 508 of the Rehabilitation Act (22) and the Web Content Accessibility Guidelines (23) exist to prevent, and which the literature on public-sector web content has repeatedly documented (24). Washington’s official distribution channel, however, publishes the manual as structured HTML (25), and it is that authoritative version which was retrieved and analyzed (06_fetch_html_manuals.py). Two consequences follow. First, page-based retention statistics are undefined for this document and for its Spanish counterpart, so the retention table covers eighteen of the twenty corpus documents. Second, and more importantly, the extraction path for this document differs from every other document in the corpus: HTML extraction yields a clean prose stream without the page furniture, running headers, and column-bleed artifacts that PDF extraction introduces. Because this document is the corpus’s best-scoring text and carries interpretive weight in Section 4, the possibility that its advantage is partly an extraction effect must be taken seriously rather than set aside. Section 2.5 describes the filter designed to remove exactly those PDF artifacts from the other documents, which narrows but does not eliminate the asymmetry; Section 5 treats it as a limitation. We note that the direction of this concern is bounded: the Washington text’s mean sentence length of 10.6 words is roughly half the corpus mean, a gap far larger than the filter’s effect on any other document.

We do not characterize Washington’s manual as inaccessible. The scanned mirror is inaccessible; the state’s own channel is not.

### 2.5 Sentence segmentation, artifact filtering, and the long-sentence audit

Sentence segmentation used spaCy (en_core_web_sm). Sentence length was counted in non-punctuation tokens.

#### 2.5.1 Why filtering is necessary

Flesch–Kincaid Grade Level and the Gunning Fog Index both take words per sentence as a direct input. Any extraction artifact that concatenates non-prose content into a single apparent sentence, whether a run-together bullet list, a flattened table row, a phone directory or a multi-column page read straight across, therefore inflates estimated difficulty. Whether very long sentences in these documents are genuine sentences or extraction failures is therefore a question to be settled empirically rather than assumed, and settling it required auditing them.

#### 2.5.2 Complete audit of long sentences

Every sentence segmented at 80 or more tokens was extracted in full and classified as genuine connected prose or as one of several artifact types (contact directory, run-together bullet list, flattened numeric table, low alphabetic content, absence of function words, fragmented-token column bleed, heading run-on, absence of a finite verb). The audit is a complete census of sentences meeting the threshold, not a sample.

Across the twenty corpus documents, 417 sentences met the 80-token threshold and all 417 were audited. Overall, 200 (48.0%) were classified as extraction artifacts and 217 (52.0%) as genuine connected prose. The most common artifact type was the run-together bullet list (83 cases, 19.9%), followed by list or table fragments lacking function words (40, 9.6%), heading run-ons (24, 5.8%), absence of a finite verb (15), multi-column bleed (15), low alphabetic content (9), flattened numeric tables (9), and contact directories (5).

The aggregate figure conceals the finding that matters, which is that the two tiers of the corpus behave in opposite ways (see Table 1).

**Table 1:** Complete audit of sentences at or above 80 tokens, by corpus tier.

Subset	Flagged	Artifact	Genuine	Artifact rate
Worker-facing guides (English)	79	54	25	68.4%
FDA Food Code 2022	306	114	192	37.3%
Spanish-language documents	32	32	0	100%
All documents	417	200	217	48.0%

All 417 qualifying sentences were classified; this is a census rather than a sample.

In the worker-facing guides, long segmented sentences are predominantly not sentences: roughly two-thirds are run-together bullet lists, flattened tables, or directory content, and the true rate is likely higher still, since bullet lists whose glyphs are dropped during extraction retain enough connectives to pass a rule-based check. Filtering is therefore warranted for this tier.

In the federal Food Code the opposite holds. Nearly two-thirds of its 306 long sentences are authentic legal prose, extended constructions with embedded enumerations and parenthetical definitions that are a genuine feature of regulatory drafting, not an extraction failure. Their median length is 108 tokens. These are among the hardest sentences a reader of that document actually encounters, and they are real.

The Spanish-language documents produced no genuine long sentences at all; every flagged case was column bleed or list concatenation, reflecting the two-column layouts in which those translations are typically published. This is a further reason, beyond formula validity, for treating them separately (Section 2.7).

Classification was rule-based and applied uniformly, with the decision features recorded for every sentence so that the classification can be re-derived or contested; the full annotated set is deposited.

#### 2.5.3 The two filter rule sets

Two extraction rule sets are reported:

The permissive rule set applies page filtering as in Section 2.4 and discards sentences only above 400 tokens. It is the least interventionist option and is reported so that the effect of the further filtering below is visible.

The strict rule set additionally targets the artifact classes the audit identified. Lines are split on bullet glyphs; a line is discarded if fewer than 65% of its characters are alphabetic, if more than 15% are digits, if fewer than 12% of its tokens are function words, or if more than 35% of its tokens are two characters or shorter; and the sentence ceiling is tightened from 400 to 60 tokens.

Each threshold corresponds to an artifact class observed in the audit rather than to a general convention: the alphabetic floor and digit ceiling remove flattened tables and numeric charts, the function-word floor removes list fragments that lack connective tissue, and the short-token ceiling removes multi-column bleed, in which text read across columns produces a stream of truncated tokens. The numeric values were set by inspection of the audited sentences and are not derived from an external standard; we report both rule sets in full for this reason, and Section 3 shows that the study’s principal conclusion is unchanged under either.

##### 2.5.3.1 These thresholds were not pre-specified, and we state so plainly

The strict rule set was designed in response to the audit and could not have been specified before it, since the audit is what identified the artifact classes the filters target. No *post hoc* justification converts an exploratory choice into a confirmatory one. What protects the conclusion is therefore not pre-specification but the reporting of every rule set, of the audit that motivated the filtering, and of a sensitivity analysis across the parameter that most affects the results (Section 2.6.3).

The strict rule set is the primary analysis and the permissive rule set is reported throughout as a sensitivity check. The rationale is that the strict stream is the one that actually consists of prose, which is what a readability formula is defined over. The direction of the adjustment is worth noting because it runs against the study’s own thesis: removing simple bullet lists leaves denser connected prose, so most guides score as *more* difficult under strict filtering, not less.

#### 2.5.4 The sentence ceiling deletes authentic text, asymmetrically

The 60-token ceiling discards genuine long sentences along with artifacts, and the audit shows it does so very unevenly. Of the 217 sentences classified as genuine prose, 192 belong to the FDA Food Code and 25 to the guides. Their mean length is 123.5 tokens and their median 108. The ceiling therefore removes from the federal document a large body of authentic regulatory prose, the hardest text in the corpus, and the text most characteristic of the document’s drafting style, while removing from the guides a comparatively small amount of genuine material along with a much larger volume of junk.

Two consequences follow, in opposite directions:

The first is conservative and favours caution about our own claims: to the extent that genuine long sentences are removed from the guides, measured difficulty of the guides is understated, so the finding that they exceed plain-language targets is if anything strengthened.

The second is not conservative and must be disclosed. Because the deletion falls overwhelmingly on the federal document, tightening the ceiling narrows the federal-versus-guides sentence-length gap by construction. The gap falls monotonically from 3.98 words at a 400-token ceiling to 1.86 words at 60 tokens (Section 2.6.3). A reader could reasonably ask whether the narrowing we report is a property of the documents or of our filter. Part of it is the filter. We therefore report the sensitivity curve rather than a single figure, and we do not rest any claim on the magnitude of that gap.

The effect-size conclusion is nonetheless robust to this choice, which is the reason it can still be reported: Cliff’s delta for the federal-versus-guides sentence-length contrast is 0.06, negligible, computed at the *permissive* 400-token ceiling, where no genuine sentence is removed. The withdrawal of the previously published medium effect size therefore does not depend on the strict ceiling and is not an artifact of it.

### 2.6 Measures and statistical analysis

#### 2.6.1 Metric selection

Metric selection follows from the cognitive account set out in Section 1 rather than from convention. Cognitive Load Theory holds that working memory is capacity- limited and that comprehension fails when intrinsic load, the irreducible difficulty of the material, is compounded by extraneous load imposed by presentation. Food safety content carries substantial intrinsic load for a novice reader: temperature thresholds, time limits, pathogen names, and conditional rules that must be held simultaneously. The measurable textual properties that add extraneous load on top of it are sentence length, which determines how much must be held in working memory before a proposition closes, and lexical difficulty, which determines how much decoding effort competes with comprehension.

Each reported metric is selected because it indexes one of those two channels:

- Mean sentence length (words per sentence), direct index of the syntactic span a reader must hold open.
- Flesch–Kincaid Grade Level (26, 27), combines sentence length and syllables per word; the standard against which the plain-language target is expressed.
- Gunning Fog Index (28), combines sentence length with the incidence of polysyllabic words, weighting lexical load more heavily than FKGL.
- SMOG Index (29); polysyllabic-word based, reported because it is the formula most used in the health-materials literature and permits comparison with it.
- New Dale-Chall score (30); the only metric here that uses a reference word list rather than syllable counting, so it indexes vocabulary familiarity rather than word length.
- Lexical density, content words as a proportion of all words; indexes propositional packing independently of sentence length.

Because Flesch–Kincaid, Gunning Fog, and SMOG all take word or syllable length and sentence length as inputs, they are not independent measures and are not treated as convergent evidence. Close agreement among them is an arithmetic consequence of those shared inputs and carries no independent information, so no concordance analysis is reported.

#### 2.6.2 Unit of analysis and inference

##### 2.6.2.1 The unit of analysis is the document

Each document contributes one value per metric. Sentences within a document are not independent observations: they share an author, a house style, an intended audience and an editorial process, so treating them as independent would understate standard errors and would describe a population of sentences rather than the documents about which this study makes claims. No sentence-level significance test is reported.

The principal analysis tests whether worker-facing guides exceed established plain-language targets. For each of the two target-referenced metrics, the document-level values were compared against the target constant (Flesch–Kincaid 6.0; Gunning Fog 8.0) using the exact one-sample Wilcoxon signed-rank test. The alternative is directional; the substantive question is whether materials are *harder* than the target, not whether they differ from it; and the direction was fixed in advance by the study’s hypothesis. Exact rather than asymptotic *p*-values are reported because *n* = 14 is well below the normal approximation’s comfortable range; readers should note that the exact test at this sample size has a discrete floor, so the smallest attainable *p*-value is bounded by *n* rather than by the strength of the effect. Mean values are accompanied by bias-corrected and accelerated (BCa) bootstrap 95% confidence intervals, 10,000 resamples, with the acceleration estimated by jackknife. BCa was chosen over the percentile interval because *n* = 14 is small and the document-level distributions are skewed, one document sits roughly three grade levels below the remainder, giving sample skewness near −1 under the primary rule set, conditions under which the percentile interval is known to be biased. Because BCa’s acceleration is itself estimated from 14 points, both intervals were computed and are deposited; they agree to within 0.2 grade levels for every reported mean, so no conclusion in this paper depends on the choice. The count of documents meeting each target is reported alongside the test, and is the more directly interpretable quantity.

Two robustness checks accompany the principal analysis: the same tests under the permissive rule set, and the count of documents meeting each target under relaxed thresholds (Flesch–Kincaid 6, 7, and 8; Gunning Fog 8, 10, and 12).

#### 2.6.3 What the inferential statistics do and do not assume

The corpus is not a probability sample. It was assembled by a documented systematic search rather than by random selection from an enumerated list, and Section 2.2.2 states that regime classification is complete for only 8 of 51 jurisdictions. The *p*-values and intervals reported here therefore require an explicit statement of what they assume, because a design-based interpretation is not available.

We adopt a model-based rather than a design-based framework. The 14 guides are treated as realizations of a document-generating process: the drafting practices by which U.S. public health authorities produce worker-facing food safety instruction. Under this framing the quantity of interest is a property of that process rather than of a finite population of documents, the tests ask whether a process producing documents like these is one that systematically exceeds the plain-language targets, and the confidence intervals describe the precision with which the process mean is estimated from 14 realizations. Model-based inference of this kind is standard where a complete enumeration is impossible and the units are not sampled at random, and it is the framework under which the reported statistics should be read.

The assumption this framework requires is exchangeability: that the 14 documents are not systematically different, in the properties measured, from other documents the same process produces. That assumption cannot be verified here, and there are identifiable reasons it might fail. Publisher jurisdictions might draft more carefully than delegator jurisdictions would if they published, in which case the corpus understates difficulty; or agencies that publish openly might do so precisely because they have invested in accessible drafting, with the same effect. Both possibilities bias in the same direction, which is conservative with respect to the claims made here, but neither is testable with these data.

We therefore report the descriptive result first and treat it as primary: 13 of the 14 guides exceed the Flesch–Kincaid target, under all three extraction rule sets and at every relaxed threshold tested. That statement is a complete enumeration of the analysed corpus and requires no sampling assumption at all. The inferential quantities are reported as a secondary characterization, valid under the model stated above, and no conclusion in this paper rests on them alone.

#### 2.6.4 The federal comparison is descriptive, and why

The corpus contains exactly one federal document. Document type is therefore completely confounded with document identity, and no test can distinguish a property of regulatory texts from a property of the 2022 Food Code. The federal-versus-guides comparison is accordingly reported descriptively, with no inferential statistic. Pooling sentences to obtain a large nominal sample would manufacture precision without adding information about either population.

For the same reason the sentence-length effect size requires careful handling. Cliff’s delta is reported as a descriptive summary of distributional overlap only. Its value is sensitive to the sentence ceiling, and that sensitivity is reported rather than resolved by a single choice: as the ceiling is tightened from 400 tokens to 60, the federal-minus-guides gap in mean sentence length falls monotonically from 3.98 words to 1.86. Any single reported effect size for this contrast is thus conditional on an analyst’s choice of ceiling, and we report the full sensitivity curve so that readers can see the dependence rather than inherit one point from it.

### 2.7 Spanish-language documents

Five Spanish-language counterparts of English documents from the same issuing jurisdictions were retrieved, integrity-checked and deposited, but are excluded from all readability analysis. Flesch–Kincaid and Gunning Fog are calibrated on English syllable and word-length distributions, so applying them to Spanish text yields numbers without interpretation, and differencing those numbers against English scores yields a quantity that does not exist. A dedicated analysis would require Spanish-validated instruments such as Fernández-Huerta or Szigriszt-Pazos interpreted on the INFLESZ scale, and a Spanish segmentation model rather than the English one used here for corpus characterization. No cross-language claim is made anywhere in this paper.

### 2.8 Reference standard for plain-language targets

No comparison is made against text written by the investigators. A short researcher-authored passage is an unreliable reference point: Flesch–Kincaid and Gunning Fog are unstable on texts of a few dozen words, where a single long sentence can move the score by several grade levels, and a passage composed for the purpose cannot be reproduced by others.

Two reference points replace it, neither of which depends on text written by the investigators.

The first is the published plain-language targets themselves. A sixth-grade reading level is the standard recommendation for public-facing health communication, and the Gunning Fog target of 8 is its counterpart in that literature. These are the criteria against which the documents are tested in Section 3.

The second is empirical and internal to the corpus. The Washington State Food Worker Manual is an actually existing, jurisdiction-issued, legally distributed food safety training document that meets both targets. It functions as an existence proof: it establishes that the targets are attainable for this content domain and this audience without requiring the investigators to demonstrate attainability by writing a specimen themselves. Its status as a reference point is observational, not experimental; it was identified by the analysis rather than selected in advance; and Section 4 draws on it accordingly.

### 2.9 Reproducibility

The retrieval harness, candidate URL list, retrieval log with per-object hashes, analysis pipeline, validation report, complete long-sentence audit, and both filter rule sets are deposited. Documents themselves are redistributed only where their licence permits; where it does not, the deposited log carries the SHA-256 digest so that a reader who retrieves the document independently can verify byte identity with the copy analyzed here.

## 3 Results

### 3.1 Corpus

The analyzed corpus comprised 20 documents retrieved from federal, state, and county sources. Fifteen were English-language: 14 worker-facing study guides and the 2022 FDA Food Code. A further five were Spanish-language counterparts of English guides from the same issuing jurisdictions; these were retrieved and deposited but are not analyzed here, because English readability formulas are not validated for Spanish text (Section 2.7).

The worker-facing guides span 11 jurisdictions across eight states: Alaska, Idaho, Oregon, Washington, California, Nevada, Oklahoma, and Virginia. Most are issued directly by a state or county health authority. Two are not, and are included because they are offered to workers as preparation for a state requirement and are freely retrievable: the Oregon State University extension guide and the Oregon eFoodcard study guide (Section 2.2.3).

Retrieval followed a documented protocol recording, for every attempt, the source URL, UTC timestamp, HTTP status, final URL after redirects, content type, payload size, SHA-256 hash, PDF structural validity, and page count. The complete retrieval log is provided in the deposited materials.

Among PDF-sourced documents, page filtering retained 479 of 508 pages across the guides and 663 of 668 pages of the Food Code. The Washington manual was obtained as structured HTML from the state’s official channel rather than as a PDF, so page-based retention is undefined for it (Section 2.4).

Corpus volume depends on the extraction rule set, and is reported for all three (see Table 2).

**Table 2:** Corpus volume under each of the three extraction rule sets, for the worker-facing guides and the 2022 FDA Food Code.

Rule set	Guide words	Guide sentences	Food Code words	Food Code sentences
Permissive	105,889	6,200	232,363	11,164
Strict	76,284	4,036	175,613	5,859
Classified	57,628	2,594	141,682	4,033

#### 3.1.1 Population governed by the analysed documents

The corpus is small in document count but not in reach. Four documents are issued at state level and apply statewide: Alaska (740,133 residents), Idaho (2,001,619), Oregon (4,272,371) and Washington (7,958,180). The remainder are issued by county or district health authorities and apply within their jurisdictions: San Diego County, California (3,298,799), Orange County, California (3,165,820), Riverside County, California (2,529,933), Clark County, Nevada (2,398,871), Rogers County, Oklahoma (101,371) and the Lord Fairfax Health District, Virginia (approximately 242,000). Pacific County, Washington, is counted within the Washington state total rather than separately.

**Figure 1:**
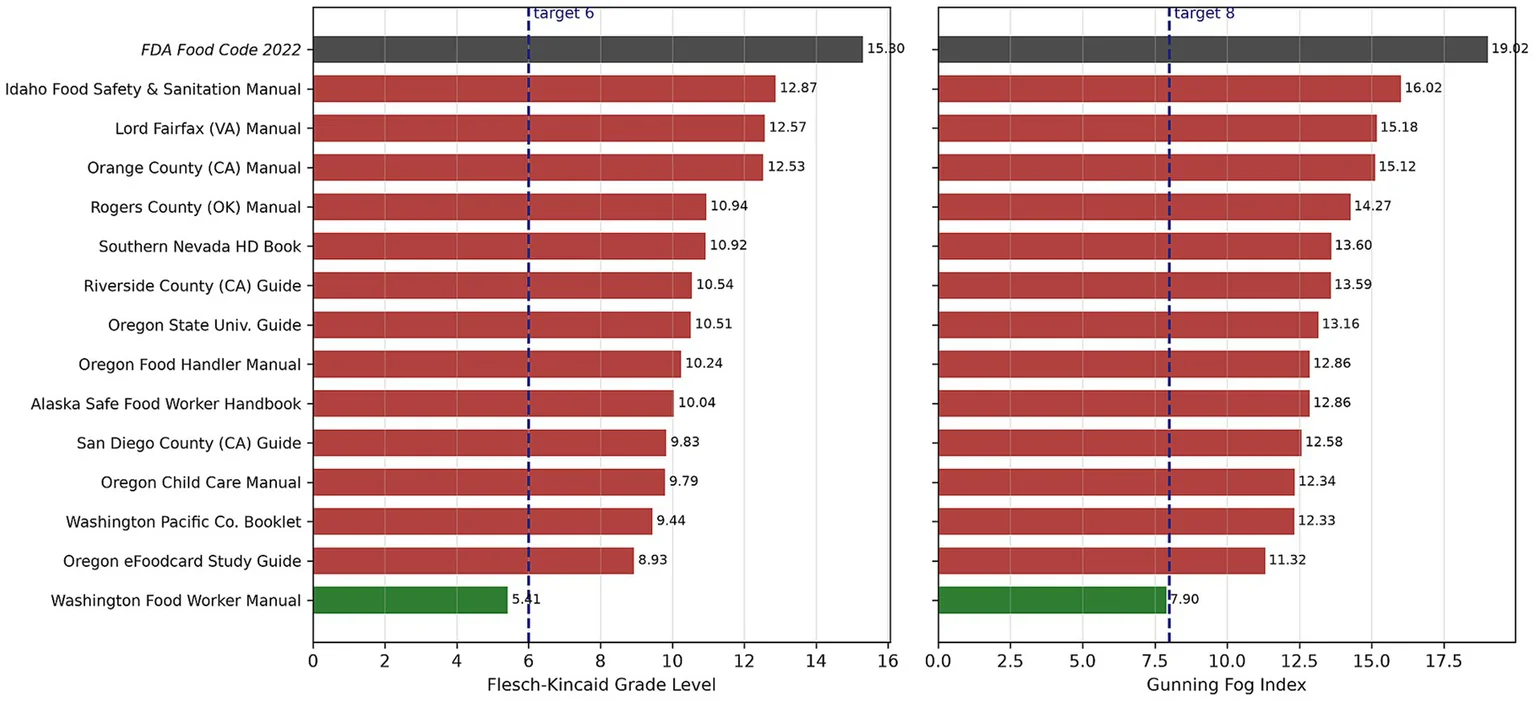
Document-level Flesch–Kincaid Grade Level and Gunning Fog Index for 14 worker-facing food handler guides and the 2022 FDA Food Code, under the primary (strict) extraction rule set. Dashed lines mark the plain-language targets of 6.0 and 8.0. The federal Food Code is shown in grey and is not pooled with the guides.

Together these jurisdictions contain approximately 26.7 million residents, or about 8 percent of the U.S. population. Population figures are U.S. Census Bureau Vintage 2024 estimates for 1 July 2024, except the Lord Fairfax Health District, for which the Virginia Department of Health’s published district population is used. These figures describe the populations to whom the analysed documents are addressed; they are not an estimate of the number of food workers, which is smaller and which we did not attempt to derive (see Figure 1).

### 3.2 Worker-facing guides exceed plain-language targets

Taking the document as the unit of analysis (*n* = 14 guides), the guides exceed both plain-language targets under every extraction rule set tested (see Table 3).

**Table 3:** Document-level readability of the 14 worker-facing guides against plain-language targets, under each of the three extraction rule sets.

Metric	Permissive	Strict	Classified
FKGL mean	10.12	10.33	11.13
95% CI (BCa)	9.04–11.59	9.15–11.11	9.84–11.97
Median	9.64	10.38	11.06
Range	5.88–16.31	5.41–12.87	5.67–14.41
Meeting FKGL ≤ 6	1/14	1/14	1/14
Mean exceedance	4.12	4.33	5.13
Wilcoxon *p*	1.22 × 10^−^⁴	1.22 × 10^−^⁴	6.11 × 10^−^⁴
GFI mean	12.81	13.08	13.94
95% CI (BCa)	11.69–14.17	11.85–13.91	12.59–14.84
Median	12.38	13.01	13.76
Range	8.37–18.24	7.90–16.02	8.27–17.23
Meeting GFI ≤ 8	0/14	1/14	0/14
Mean exceedance	4.81	5.08	5.94
Wilcoxon *p*	6.10 × 10^−5^	6.11 × 10^−^⁴	6.10 × 10^−5^

FKGL, Flesch-Kincaid Grade Level; GFI, Gunning Fog Index; CI, confidence interval; BCa, bias-corrected and accelerated bootstrap.

Thirteen of the 14 guides exceeded the Flesch–Kincaid target of 6, under all three extraction rule sets. At most one met the Gunning Fog target of 8, and under two of the three rule sets none did. These counts are a complete enumeration of the analysed corpus and rest on no sampling assumption. One-sample exact Wilcoxon signed-rank tests against each target constant, one-sided, were significant at *p* < 0.001 in every case; as Section 2.6.3 sets out, those tests and the accompanying intervals are model-based rather than design-based, and should be read as characterizing the document-generating process under an exchangeability assumption that the corpus cannot verify.

Because the unit of analysis is the document rather than the sentence, these quantities are unaffected by the clustering of sentences within documents, and they concern the documents themselves rather than the sentences of any particular document.

The conclusion is not sensitive to the choice of target. Relaxing the Flesch–Kincaid threshold from 6 to 7 or to 8 leaves the same single guide compliant under all three rule sets. Relaxing the Gunning Fog threshold from 8 to 10 admits at most one document, and at 12 admits one further document under the classified rule set, two under strict, and five under permissive.

Mean sentence length across the guides was 20.07 words under permissive rules (95% CI 18.00–21.38), 19.19 under strict (17.09–20.36), and 25.39 under classified (22.63–27.42), the last being higher because classification removes short list items along with other non-prose material (Section 2.5).

All confidence intervals are bias-corrected and accelerated (BCa) bootstrap intervals, 10,000 resamples. Percentile intervals were also computed and are deposited; the two methods agree to within 0.2 grade levels for every mean reported above, so no conclusion depends on the choice (Section 2.6.2). Per-document values under all three extraction rule sets are given in Supplementary Table S1, and the full classification of all 417 audited sentences is given in Supplementary Table S2.

### 3.3 One jurisdiction demonstrates that the target is largely attainable

The Washington State Food Worker Manual is the least demanding document in the corpus by a wide margin, and the only one to meet a plain-language target (see Table 4).

**Table 4:** Readability of the Washington State Food Worker Manual under each of the three extraction rule sets, the only document in the corpus to meet a plain-language target.

Metric	Permissive	Strict	Classified
FKGL	5.88	5.41	5.67
Gunning fog	8.37	7.90	8.27
Mean sentence length	11.35	10.55	13.06

It meets the Flesch–Kincaid target of 6 under all three rule sets. It meets the Gunning Fog target of 8 under one of the three, missing it narrowly under the other two (8.37 and 8.27 against a target of 8). We state the result this way rather than as unqualified compliance because the Gunning Fog outcome depends on an analytic choice, and the qualification does not weaken the substantive point: the next least demanding guide scores 9.69 to 8.01 on Flesch–Kincaid and 12.18 to 10.58 on Gunning Fog, so Washington is separated from the rest of the corpus by roughly three grade levels regardless of rule set.

It achieves this while covering the same content domains as the other guides: foodborne illness, worker health, handwashing, bare-hand contact, temperature control, cooling, cross-contamination, allergens, sanitizing, and cleanup.

One structural feature of the document is worth reporting here because it bears directly on interpretation. Washington instructs very largely through short list items rather than connected paragraphs: 63% of its segmented sentences were classified as list or fragment content, the highest proportion in the corpus, against a corpus average of 38%. Under the classified rule set, which measures connected prose only, that design choice is invisible; the document is scored on the 37% of its text that is continuous prose. This is why its Gunning Fog value rises above the target under that rule set. The list-based structure is not a defect to be filtered away; it is a recognized plain-language technique for reducing extraneous cognitive load, and Section 4 treats it as one of the document’s principal design virtues.

This is an existence proof rather than an outlier to be explained away. It establishes that the reading burden documented across the remainder of the corpus is a property of drafting choices rather than of food safety content itself.

### 3.4 The federal Food Code is substantially harder than the worker guides

The 2022 FDA Food Code is the most demanding document in the corpus under every rule set, and by a widening margin as artifact filtering becomes more rigorous (see Table 5).

**Table 5:** Readability of the 2022 FDA Food Code compared with the most demanding worker-facing guide, under each of the three extraction rule sets.

Measure	Permissive	Strict	Classified
FKGL	12.98	15.30	16.97
Gunning Fog	16.46	19.02	20.75
Mean sentence length	23.48	22.30	38.87
Hardest guide (FKGL)	16.31 (Rogers Co.)	12.87 (Idaho)	14.41 (Lord Fairfax)

The separation between the regulatory tier and the instructional tier is real and in the expected direction: the document written for inspectors and compliance officers is harder than the documents written for workers. The concern is not that the two tiers differ. It is that the more accessible tier still sits four to five grade levels above the recommended ceiling (Section 3.2).

#### 3.4.1 Sentence-length contrast and effect size

Under the classified rule set, the only one in which both text streams are cleaned symmetrically and no sentence-length ceiling is imposed, the Food Code’s mean sentence length is 38.87 words against 25.00 for the pooled guides, a difference of 13.87 words, with Cliff’s delta of 0.35.

This effect size is stable across every sentence ceiling tested (see Table 6).

**Table 6:** Sensitivity of the federal-versus-guides sentence-length contrast to the sentence-length ceiling, under the classified rule set. Values are mean sentence length in words; Cliff’s delta is a descriptive measure of distributional overlap.

Ceiling (tokens)	Guides (words)	Food Code (words)	Difference	Cliff’s delta
60	22.17	28.49	6.33	0.313
80	23.50	31.32	7.82	0.325
100	24.27	33.01	8.74	0.329
150	24.87	35.65	10.78	0.341
200	24.93	36.98	12.05	0.347
400 (none binding)	25.00	38.87	13.87	0.352

The mean difference is ceiling-dependent, as it must be when one document contains most of the corpus’s long sentences; the effect size is not.

The estimate does depend on whether non-prose content is removed from both streams. Under the permissive rule set, which retains headings, section numbers, and enumerated fragments in both, the Food Code’s mean sentence length is 23.48 words against 19.50 for the pooled guides, and Cliff’s delta is 0.06. That comparison sets one contaminated stream against another: the retained material is disproportionately short in both documents, and its presence compresses the two distributions toward each other, producing overlap that is a property of the extraction rather than of the writing. The classified rule set removes that material from both streams and is the appropriate basis for the estimate. The permissive value is reported here so that the dependence is visible, and it is the reason no single effect size for this contrast should be quoted without its extraction rule.

We nonetheless report this contrast descriptively and attach no inferential statistic to it. The corpus contains exactly one federal document, so document type is completely confounded with document identity, and no test can distinguish a property of regulatory texts in general from a property of the 2022 Food Code in particular. Pooling sentences across documents to obtain a large nominal sample would treat clustered observations as independent and manufacture precision without adding information about either population. The Mann–Whitney No inferential test is therefore reported for this contrast.

One document-level value in the corpus is worth flagging because it is sensitive to artifact filtering. The Rogers County (OK) manual scores FKGL 16.31 under the permissive rule set, which would place it above the federal Food Code, but falls to 10.94 under strict filtering and 11.46 under classification-based filtering. The document contains exactly one sentence at or above the 80-token flag, so the permissive value is driven by page-level non-prose content rather than by long sentences. We therefore do not report any worker-facing guide as more demanding than the federal Food Code.

### 3.5 Sentence-length distributions

**Figure 2:**
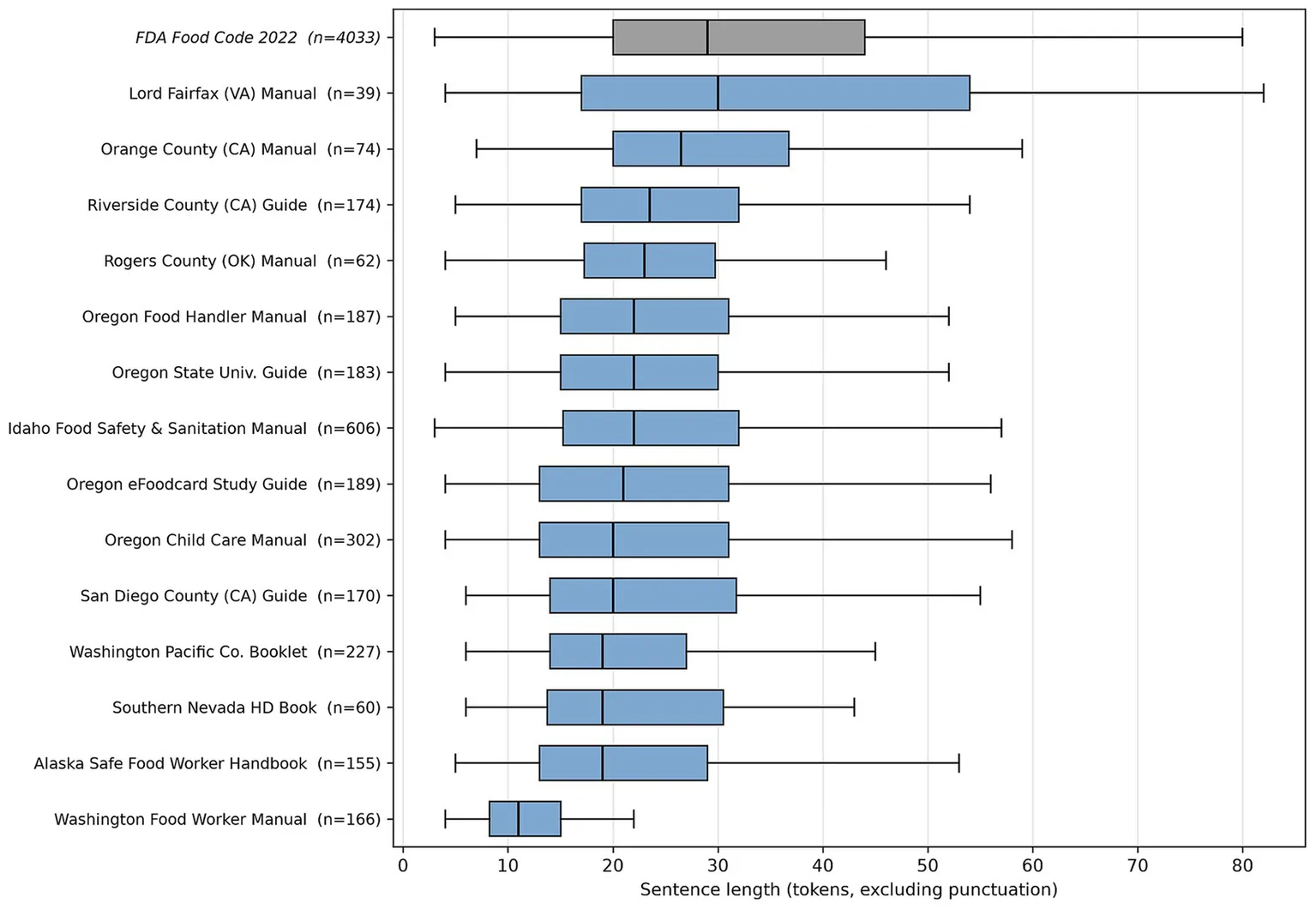
Per-document sentence-length distributions under the classification-based rule set. Boxes show the median and interquartile range; outliers are suppressed. Retained sentence counts are given on the axis, since documents differ by two orders of magnitude in retained sentences. Distributions are shown per document rather than pooled.

Sentence-length distributions are reported descriptively and per document (Figure 2) rather than pooled, which reveals variation the pooled presentation obscured: guide mean sentence lengths range from 13.1 to 35.0 words under the classified rule set, and the interquartile ranges of the least and most demanding guides do not overlap.

Per-document presentation also discloses a qualification that the document-level means conceal. The Lord Fairfax (VA) manual has a median sentence length and an interquartile range exceeding those of the Food Code, so the federal document is not the most demanding text in the corpus on every sentence-length statistic. It is the most demanding on the mean, and on both readability indices. The Lord Fairfax figure rests on 39 retained sentences, the smallest retained sample in the corpus, against 4,033 for the Food Code, and per-document sentence counts are shown in Figure 2 for this reason. We do not treat the comparison as informative at that sample size, and note it here rather than allowing a reader to discover an apparent contradiction in the figure.

The complete long-sentence census (Section 2.5.2) is relevant to reading this figure. Of 417 sentences segmented at 80 or more tokens across the corpus, 48% were extraction artifacts and 52% genuine prose, but the split differs sharply by tier: 68.4% artifacts among the worker guides against 37.3% for the Food Code. Long segments in the guides are predominantly run-together bullet lists and flattened tables; long sentences in the Food Code are predominantly authentic legal constructions with a median length of 108 tokens.

### 3.6 Reference standard

No comparison against researcher-authored text is reported, for the reasons given in Section 2.8.

Two reference points are used instead. The first is the published plain-language targets themselves, against which every document is tested in Section 3.2. The second is internal to the corpus: the Washington State Food Worker Manual is an existing, jurisdiction-issued, legally distributed training document that meets the Flesch–Kincaid target under every rule set tested, and therefore establishes attainability empirically rather than by demonstration.

## 4 Discussion

### 4.1 Principal findings

Fourteen publicly available worker-facing food safety training documents were analyzed at the document level. One met the Flesch–Kincaid target of sixth-grade reading level; at most one met the Gunning Fog target of 8. The mean guide sat between four and five grade levels above the recommended ceiling depending on extraction rules, and the result held under every rule set and every relaxation of the target threshold tested. The federal Food Code was harder than every guide, with sentences averaging roughly 39 words against 25 for the guides.

The interpretive question is what those numbers mean, and the answer is not that workers cannot understand food safety. It is narrower and more useful: these documents impose a measurable processing burden that is unnecessary, because one document in the same corpus, covering the same content for the same audience, does not impose it.

### 4.2 Cognitive load theory as the interpretive frame

Cognitive Load Theory holds that working memory is severely capacity-limited for novel information, and that instructional failure occurs when the total load imposed by material exceeds that capacity (8, 9, 31). The theory distinguishes load intrinsic to the material from load imposed extraneously by how the material is presented, and it makes a directional claim that this study is designed around: extraneous load is the component under the author’s control, and reducing it frees capacity for learning.

Food safety instruction carries substantial intrinsic load. A worker must hold simultaneously a set of numeric thresholds (holding temperatures, cooling intervals, sanitizer concentrations), a set of conditional rules whose antecedents are themselves technical (what counts as a ready-to-eat food, when bare-hand contact is permitted, which illnesses require exclusion rather than restriction), and a causal model connecting pathogens to practices. That load is irreducible; it is what the training is for. It is also being carried, in many cases, by readers who are new to the occupation, reading in a second language, or reading under time pressure before a required examination.

This is why the metrics in this study were selected as they were, rather than because they are conventional. Each indexes a channel through which drafting choices add extraneous load on top of that intrinsic burden:

Sentence length indexes how long a proposition must be held open before it resolves. A 39-word regulatory sentence with embedded parenthetical definitions requires the reader to maintain an incomplete syntactic structure across several subordinate clauses while simultaneously decoding technical vocabulary. The working-memory cost is not a metaphor; it is the mechanism CLT describes.

Lexical difficulty, indexed here by Gunning Fog’s polysyllabic count and by Dale-Chall’s reference-word list, indexes decoding effort. Every word that must be resolved effortfully rather than recognized competes for the same limited capacity that comprehension requires.

Lexical density indexes propositional packing: how much content is delivered per unit of text, independent of sentence length.

Because the theory locates the remediable problem in extraneous load, it implies that comparable content should be deliverable at lower processing cost without simplifying the content itself. The corpus cannot test that implication, since testing it would require measuring what readers do. What the corpus can show is whether documents in this domain vary widely in the textual features the theory identifies, and whether any document achieves the recommended levels while addressing the same subject matter.

#### 4.2.1 Washington as the test case

The Washington State Food Worker Manual meets the Flesch–Kincaid target under every extraction rule set, with a mean sentence length of 11 to 13 words against a corpus mean of 19 to 25. It addresses the same content domains as the other guides: foodborne illness, worker health and exclusion, handwashing, bare-hand contact, temperature control, cooling, cross-contamination, allergens, sanitizing and cleanup. Coverage of the same domains does not establish that the documents are equivalent in the volume of material, technical detail, exceptions, worked examples or instructional depth they provide, and we have not measured those properties. What can be said is that a document addressing this subject matter for this audience reaches the recommended level, which the remaining thirteen do not.

Two drafting choices distinguish it, and both are exactly what CLT prescribes:

The first is segmenting: it delivers instruction in short, self-contained units rather than in continuous exposition. Sixty-three percent of its segmented sentences are list items or short fragments, the highest proportion in the corpus, against an average of 38%. This corresponds to what cognitive load theory calls segmenting: each proposition closes before the next begins, so a reader is not required to hold partial structures across clause boundaries. We report the structural feature; the inference to working memory is the theory’s, not a measurement of ours.

This finding has a methodological corollary worth stating plainly, because it cuts against our own primary analysis. The extraction rule set that isolates connected prose scores Washington *worse* on Gunning Fog than the permissive rule set does, because it discards the list-based instruction that constitutes most of the document. A readability formula applied to the prose remainder measures what is left after the document’s principal plain-language technique has been filtered out. We report the result under all three rule sets for this reason, and the divergence is itself informative: list structure is not noise in the measurement, it is a design feature of the document.

The second is plain lexis in place of regulatory register. Washington’s Dale-Chall score is 8.36 against a corpus range extending to 10.95 for the hardest guide and 12.11 for the Food Code (Table 7), indicating substantially greater reliance on high-frequency vocabulary.

**Table 7:** Readability of U.S. food handler training materials and the federal food code (primary analysis, strict rule set).

Document	Tier	Words	Sentences	Mean sent. length	FKGL	Gunning Fog	SMOG	Dale-Chall	Lexical density (%)
Washington Food Worker Manual	State	4,435	444	10.55	5.41	7.90	9.08	8.36	60.99
Oregon eFoodcard Study Guide	State	5,250	259	19.31	8.93	11.32	11.48	9.37	56.52
Washington Pacific Co. Booklet	County	5,954	314	17.65	9.44	12.33	11.94	9.45	58.13
Oregon Child Care Manual	State	9,107	463	19.08	9.79	12.34	11.77	9.46	55.34
San Diego County (CA) Guide	County	5,008	241	19.27	9.83	12.58	12.40	10.02	57.67
Alaska Safe Food Worker Handbook	State	4,902	298	16.60	10.04	12.86	12.09	9.63	56.08
Oregon Food Handler Manual	State	5,585	256	19.91	10.24	12.86	12.43	9.59	57.64
Oregon State Univ. Guide	State	6,352	360	18.57	10.51	13.16	12.94	10.45	57.24
Riverside County (CA) Guide	County	4,520	218	22.53	10.54	13.59	13.10	9.72	55.24
Southern Nevada HD Book	County	2,347	127	18.15	10.92	13.60	13.08	10.90	63.48
Rogers County (OK) Manual	County	1,491	73	22.97	10.94	14.27	13.63	9.84	55.26
Orange County (CA) Manual	County	2,644	124	21.78	12.53	15.12	14.12	10.71	58.96
Lord Fairfax (VA) Manual	County	1,556	62	21.52	12.57	15.18	14.33	10.89	63.30
Idaho Food Safety & Sanitation Manual	State	17,133	797	20.77	12.87	16.02	14.96	10.95	58.51
*FDA Food Code 2022*	Federal	175,613	5,859	22.30	15.30	19.02	16.90	12.11	57.70
**Guide mean (*n* = 14)**				**19.19**	**10.33**	**13.08**	**12.67**	**9.95**	**58.17**

Targets: Flesch–Kincaid ≤ 6.0, Gunning Fog ≤ 8.0. Documents ordered by Flesch–Kincaid; the federal Food Code is listed last and is never pooled with the guides.

Bold values in the final row are means across the 14 worker-facing guides. The FDA Food Code is a federal regulation rather than a worker-facing guide and is excluded from these means.

This document is what makes the paper’s recommendations concrete rather than exhortatory. It shows that the reading levels recommended for public-facing health communication are attainable in this domain, which is a weaker claim than saying the burden elsewhere is caused by drafting choices, but a sufficient one: an agency revising its materials has a worked example rather than an aspiration. Whether the drafting features described below in fact reduce the burden on readers is a question for direct testing, not for textual analysis.

#### 4.2.2 What the regulatory tier does and does not show

The federal Food Code is the hardest document in the corpus by a wide margin, and its sentence-length distribution is separated from the guides’ with a Cliff’s delta of approximately 0.35, stable across every analytic choice tested.

This is the expected direction and is not in itself a criticism. The Food Code is a model regulation addressed to regulators, drafted for legal precision and adoption by reference; its readers are inspectors, attorneys, and operators, not food handlers. Precision of that kind has genuine costs in readability that are justified by its function.

The finding that matters is the relationship between the tiers. The instructional tier exists to translate the regulatory tier for a different audience. Measured against that purpose, the translation is incomplete: the guides are easier than the Code, but they remain four to five grade levels above the level recommended for public-facing health communication (16, 17, 32). Translation has occurred in register but not to the point of accessibility.

### 4.3 Implications

#### 4.3.1 For the agencies that write these documents

The corpus supports concrete, testable drafting guidance rather than exhortation, because the target document already exists and can be inspected.

Sentence length is the highest-yield target: It is an input to every formula used here and the variable on which the corpus varies most (11 to 35 words). The guides that come closest to the target are those that decompose procedures into steps rather than describing them in continuous prose.

Lists are not a formatting preference; they are a load-reduction technique: Our own analysis demonstrates their effect: filtering list content out of the Washington manual raises its measured difficulty above the plain-language target that it otherwise meets. Agencies revising materials should convert conditional procedural prose into enumerated steps as a first pass.

Vocabulary substitution should be systematic rather than incidental: Dale-Chall differences across the corpus indicate that the harder guides rely on technical register where common vocabulary is available.

Formula scores should be treated as a drafting instrument, not a compliance certificate: A document can be tuned to a target score by shortening sentences without improving comprehensibility. The scores identify candidates for revision; they do not validate the revision. Section 4.4 develops this limit.

#### 4.3.2 Readability attaches to a statutory duty in at least two jurisdictions

The strongest justification for measuring these specific documents is not that readability is generally desirable. It is that in some jurisdictions the state has assumed a legal obligation with respect to them.

Washington Administrative Code 246-217-025(3) requires the local health officer to furnish every applicant with a copy of the current *Food and Beverage Service Workers’ Manual*. The state does not merely publish the document; it compels its distribution to every worker seeking a card. A statutory duty to furnish a document is, in substance, a duty premised on that document being usable by the person receiving it. Washington happens to be the jurisdiction that has drafted its manual to a sixth-grade level, but the duty and the drafting quality are independent, and the duty would be equally binding on a manual written at grade twelve.

Idaho illustrates the same relationship from the opposite direction. Idaho publishes a free worker manual without mandating worker-level certification, and state guidance advises that the answers to the food safety examination are contained in the manual (33). Where that is so, the manual’s readability may affect a worker’s ability to use it when preparing for or sitting the examination: a worker reading below the level at which the manual is written may have difficulty locating the answers it is said to contain. Examination performance was not measured in this study. Idaho’s manual scores between 12.16 and 13.43 on Flesch–Kincaid depending on extraction rules, roughly a high-school senior’s reading level, for a document whose stated function is to be searched by an entry-level worker under examination conditions.

The general implication is that jurisdictions which mandate training, publish the material, and gate employment on an examination drawn from it have assumed responsibility for the material’s usability, whether or not they have recognized it. Readability measurement is one way of auditing whether that responsibility is being discharged.

#### 4.3.3 Two existing regulatory models for accommodation

A small number of jurisdictions have already adopted alternatives to the written examination, set out in their food codes and grounded explicitly in the Americans with Disabilities Act. To our knowledge these provisions have not been described in the published literature. They are significant here because they show that the accommodation question is not hypothetical: two states have answered it, and they have answered it differently in a way that matters.

Washington issues a *limited duty food worker card* (WAC 246-217-045) (34). The local health officer may issue one where necessary to accommodate a person with a disability; no written examination is required; the applicant communicates which low-risk activities they will perform, and the approved activities are listed on the card. A separate provision requires test accommodations in accordance with the ADA (WAC 246-217-025(10)). The provision has been in force since 1999.

Alaska takes a different route (18 AAC 31.330(f)) (35). The department waives the examination requirement where necessary for ADA compliance if the worker demonstrates understanding sufficient to protect public health for the activities that the worker “performs or is expected to perform,” and lists each approved activity on the card.

Both provisions solve the immediate access problem, and both are preferable to the silence of the other jurisdictions whose rules we examined. But they differ on a dimension that the disability employment literature identifies as central.

Washington’s card is available for activities the rule characterizes as low public health risk (dishwashing, bussing, filling condiment containers) and the card itself is labelled a limited duty card (WAC 246-217-035 (5)(a)). The accommodation therefore grants examination access at the cost of an occupational ceiling, and it discloses that the holder required accommodation on a credential that employers routinely inspect. Federal compliance guidance written for this sector frames accommodation as an adjustment to how a particular job is performed, assessed against the essential functions of that job (36, 37); a credential that instead restricts its holder to a predefined category of low-risk tasks operates on a different principle. An accommodation that reaches the worker in the form of a visibly marked, scope-restricted credential is not straightforwardly an accommodation, and we note the tension without evidence on how the provision operates in practice.

Alaska’s provision is engineered better on precisely this axis. The waiver is scoped to the activities the worker actually performs or is expected to perform, with no presumption that those activities are low-risk ones. A worker demonstrating understanding of the requirements applicable to cooking is authorized to cook. The scope of the credential follows the worker’s job rather than an assumption about the worker’s capacity.

The recommendation that follows is specific and implementable: jurisdictions adopting an accommodation pathway should scope it to the worker’s actual occupational range rather than to a predefined low-risk category, and should avoid encoding accommodation status in the visible form of the credential. Alaska’s text is available as a drafting model.

We note the limit of this analysis. It is an audit of regulatory text, conducted against primary legal sources, in a small number of jurisdictions verified to date. It is not evidence about how these provisions operate in practice, how often limited duty cards are issued, or what happens to the workers who hold them. Those are empirical questions this study does not answer.

#### 4.3.4 The tier that cannot be audited at all

The jurisdictional taxonomy developed as this study’s sampling frame produced a finding that the readability results themselves cannot express.

Among the jurisdictions whose rules we verified, several classified as delegators mandate worker training but delegate its content to private accredited vendors. Those materials are copyrighted, priced, and distributed under licence. Their readability cannot be assessed by a researcher, an advocate, a worker, or, absent a specific contractual reservation, the delegating agency itself.

Every document analyzed in this study comes from a publisher jurisdiction. The corpus is therefore drawn from the subset of jurisdictions whose materials can be examined at all, and the finding that those materials exceed plain-language targets is a finding about the transparent tier. How many jurisdictions fall into each regime nationally is not established here; our classification is complete for eight jurisdictions. Whether privately produced materials are better or worse is unknown and, under present arrangements, unknowable.

This is an accountability gap rather than a readability finding, and it is arguably the more consequential of the two. A jurisdiction that mandates training and delegates its content has made the accessibility of a legal requirement into a matter of private discretion. The remedy does not require the state to author materials: a procurement or accreditation condition requiring that approved vendor materials meet a stated readability standard, and be available for audit against it, would place the transparent and delegated tiers on equal footing.

### 4.4 Limitations

Readability formulas do not measure comprehension: This is the central limitation and it constrains every claim above. Flesch–Kincaid, Gunning Fog, SMOG, and Dale-Chall are functions of surface features, syllables, word length, sentence length, and word-list membership. They are validated as predictors of text difficulty in aggregate, not as measures of whether a particular reader understands a particular passage. No participant read any document in this study. Where this paper says a document imposes a high processing burden, it is making a claim about the text; where it would say a worker failed to understand, it would be making a claim the design cannot support, and it does not make it.

Formula scores are also gameable: A document can be driven to a target score by mechanically shortening sentences while leaving the underlying concepts opaque, and the same score can be achieved by texts that differ substantially in clarity. The recommendations in Section 4.3 should therefore be read as identifying candidates for revision and as specifying drafting techniques with independent theoretical support, not as certifying that a document meeting a threshold is comprehensible. Direct comprehension testing with food workers is the necessary next step and is not substitutable by textual analysis.

A further step separates comprehension from outcomes: Reviews of food safety training in commercial settings report that measured knowledge gains do not reliably translate into behavioral compliance (38, 39), and that interventions addressing the barriers workers face in applying what they have learned are what shift practice (40). A more readable manual is therefore a necessary rather than a sufficient condition for safer food handling, and this study speaks only to the first link in that chain.

Claims about workers with disabilities are confined to regulatory text: An earlier version of this work framed part of its argument around workers with intellectual and developmental disabilities. That framing has been removed. The design supports no inference about how any group of workers processes these documents, and readability measurement cannot stand in for evidence about cognitive accessibility for a specific population. What survives is narrower and better grounded: an audit of what a small number of state food codes actually provide, conducted against primary legal sources, reported as regulatory analysis rather than as a claim about workers.

The sampling frame is partial: Regime classification has been verified against primary sources for eight of fifty-one jurisdictions, with document retrieval pursued additionally at county level in four states. The corpus is not a census of publisher jurisdictions and is not claimed to be one. Inference is confined to publicly available worker-facing guides.

One federal document: The regulatory tier is represented by a single text, so document type is confounded with document identity. The federal-versus-guides comparison is descriptive throughout and no inferential statistic is attached to it.

Results depend on extraction rules, and the dependence is reported rather than resolved: Three rule sets were applied. The principal finding is invariant: one of fourteen guides meets the Flesch–Kincaid target under all three, with all tests significant at *p* < 0.001. Other quantities are not invariant, the guide mean ranges from 10.12 to 11.13, and Washington meets the Gunning Fog target under one rule set of three. Readers should treat single-figure readability estimates for PDF-extracted corpora with corresponding caution; the choice of artifact filter is a researcher degree of freedom that is rarely reported and can move an estimate by more than a grade level.

One document was obtained by a different route: The Washington manual circulates as a scanned PDF with no text layer, so the state’s official HTML version was analyzed instead. HTML extraction avoids the page furniture and column artifacts that PDF extraction introduces, and this document carries substantial interpretive weight. The advantage may therefore be partly an extraction effect. Two considerations bound the concern: the artifact filters were designed to remove exactly those PDF artifacts from the comparison documents, and Washington’s separation from the rest of the corpus, roughly three grade levels; is an order of magnitude larger than the effect the filters have on any other document.

Spanish-language materials were not analyzed: Five Spanish counterparts were retrieved and deposited but excluded, because English-calibrated formulas are not valid on Spanish text and no defensible cross-language grade-level comparison is available from them. Given that these documents exist precisely to serve workers who are likely to face the greatest reading burden, their assessment with Spanish-validated instruments is a priority for subsequent work.

## 5 Conclusion

Food handler training materials published by U.S. public health authorities are written well above the reading level recommended for public-facing health communication. Of fourteen publicly available worker-facing guides analyzed at the document level, one met the sixth-grade Flesch–Kincaid target and at most one met the Gunning Fog target of 8, with the average guide sitting four to five grade levels above the ceiling. The result is invariant to the extraction rules applied, to the choice of readability formula, and to reasonable relaxations of the target itself.

The corpus itself indicates that lower reading levels are attainable. The Washington State Food Worker Manual covers the same content domains as the other guides, foodborne illness, worker health and exclusion, handwashing, bare-hand contact, temperature control, cooling, cross-contamination, allergens, sanitizing, cleanup, and meets the Flesch–Kincaid target under every analysis we ran, with sentences averaging eleven to thirteen words against a corpus average of nineteen to twenty-five. Two drafting decisions distinguish it, and any agency can copy both. We did not measure whether the documents carry identical instructional content, so the claim here is one of demonstrated attainability rather than proven equivalence.

The first is to deliver instruction in short, self-contained units rather than in continuous exposition. Nearly two-thirds of the Washington manual’s text is list-structured, the highest proportion in the corpus and roughly double the average. This is the single highest-yield change available to an agency revising its materials: converting conditional procedural prose into enumerated steps shortens sentences, which is an input to every readability formula used here, and closes each proposition before the next begins, which is what the cognitive literature predicts will reduce processing burden. The effect is large enough to be visible in our own methodology; when list structure is filtered out of the Washington manual and only its connected prose is scored, the document’s measured difficulty rises above the target it otherwise meets.

The second is to prefer common vocabulary to regulatory register wherever the regulation does not require the technical term. The spread across the corpus on the Dale-Chall scale, which measures reliance on a reference list of familiar words rather than counting syllables, runs from 8.36 for the Washington manual to 10.95 for the most demanding guide and 12.11 for the federal Food Code. Much of that distance is discretionary.

Neither recommendation requires an agency to commission new research or to compromise technical accuracy. Both can be applied to an existing document by its existing authors, and the target text is public.

Two further points follow from what jurisdictions have already committed themselves to. Washington requires its local health officers to furnish a copy of the worker manual to every applicant, and Idaho’s guidance advises that the answers to its food safety examination are contained in its manual, a manual written between the twelfth and thirteenth grade levels. Where a state compels distribution of a document, or gates employment on an examination drawn from it, the readability of that document has ceased to be a matter of good practice and has become a condition of the requirement functioning as intended. Agencies in that position should treat periodic readability assessment of their worker materials as part of maintaining the requirement, not as an optional improvement.

The same logic extends to jurisdictions that mandate training but delegate its content to private vendors. In those states the materials that satisfy a legal requirement are copyrighted and unavailable for inspection, so no assessment of their accessibility is possible by anyone outside the vendor. States in this position can close the gap without authoring materials themselves, by attaching a readability standard and an audit right to vendor accreditation.

Finally, two of the jurisdictions whose rules we examined have adopted an accommodation provision that the others have not. Alaska waives the food worker examination where necessary for compliance with the Americans with Disabilities Act if the worker demonstrates understanding sufficient for the activities they perform or are expected to perform, and lists those activities on the card. Washington issues a limited duty card requiring no written examination, scoped to activities its rule characterizes as low public health risk, and labelled as limited duty on the credential itself. Both provide access where the other jurisdictions we examined provide nothing. But they differ in a way that other states should notice before copying either: Alaska’s waiver follows the worker’s actual job, while Washington’s confines the holder to a predefined low-risk category and discloses on an employer-inspected credential that an accommodation was required. Jurisdictions drafting such a provision should scope it to the worker’s occupational range rather than to an assumption about their capacity, and should not encode accommodation status in the visible form of the card. Alaska’s text is the better model and is available to be adapted.

What this study establishes is that these documents are harder to read than they need to be, and what it cannot establish is what workers actually understand when they read them. Readability formulas measure surface properties of text, not comprehension, and a document can be tuned to a target score without becoming clearer. The drafting changes recommended here rest on textual evidence and on theory; confirming that they improve worker understanding requires testing the revised materials with the workers who must use them. That is the necessary next step, and it is now well specified: there is a corpus, a documented gap, a working model in the public domain, and two regulatory templates for the workers whom a written examination excludes.

## Data Availability

The datasets presented in this study can be found in online repositories. The names of the repository/repositories and accession number(s) can be found at: https://doi.org/10.5281/zenodo.20651801.
