# Supplementary material for: Readability of U.S. food handler training materials: a natural language processing analysis of worker study guides and the federal Food Code
Source: Front Public Health. 2026 Aug 27;14:1911335. doi: 10.3389/fpubh.2026.1911335 (PMC13558185; doi:10.3389/fpubh.2026.1911335)
Supplement: Supplementary file 1 [file Table_1.DOCX]

# Supplementary Material

Supplementary material accompanying Brako M, Naig AR, "Readability of U.S. food handler training materials: a natural language processing analysis of worker study guides and the federal Food Code".

## Supplementary Table S1. All headline quantities under all three extraction rule sets

| **Document** | **FKGL perm.** | **FKGL strict** | **FKGL class.** | **GFI perm.** | **GFI strict** | **GFI class.** | **MSL perm.** | **MSL strict** | **MSL class.** |
| --- | --- | --- | --- | --- | --- | --- | --- | --- | --- |
| Washington Food Worker Manual | 5.88 | 5.41 | 5.67 | 8.37 | 7.9 | 8.27 | 11.35 | 10.55 | 13.06 |
| Oregon eFoodcard Study Guide | 8.01 | 8.93 | 9.69 | 10.58 | 11.32 | 12.18 | 20.1 | 19.31 | 25.1 |
| Washington Pacific Co. Booklet | 9.23 | 9.44 | 10.29 | 12.01 | 12.33 | 13.2 | 19.0 | 17.65 | 24.15 |
| Oregon Child Care Manual | 8.19 | 9.79 | 11.03 | 10.85 | 12.34 | 13.67 | 21.02 | 19.08 | 25.88 |
| San Diego County (CA) Guide | 9.06 | 9.83 | 10.95 | 11.85 | 12.58 | 13.68 | 18.56 | 19.27 | 26.09 |
| Alaska Safe Food Worker Handbook | 9.52 | 10.04 | 10.95 | 12.36 | 12.86 | 13.82 | 17.2 | 16.6 | 23.55 |
| Oregon Food Handler Manual | 8.34 | 10.24 | 11.1 | 10.91 | 12.86 | 13.7 | 19.23 | 19.91 | 27.32 |
| Oregon State Univ. Guide | 9.76 | 10.51 | 11.02 | 12.39 | 13.16 | 13.49 | 19.06 | 18.57 | 23.75 |
| Riverside County (CA) Guide | 10.46 | 10.54 | 11.3 | 13.47 | 13.59 | 14.49 | 21.59 | 22.53 | 25.89 |
| Southern Nevada HD Book | 11.9 | 10.92 | 11.16 | 14.58 | 13.6 | 13.96 | 23.37 | 18.15 | 24.3 |
| Rogers County (OK) Manual | 16.31 | 10.94 | 11.46 | 18.24 | 14.27 | 14.87 | 21.96 | 22.97 | 24.74 |
| Orange County (CA) Manual | 10.72 | 12.53 | 13.31 | 13.46 | 15.12 | 16.03 | 25.0 | 21.78 | 30.23 |
| Lord Fairfax (VA) Manual | 12.21 | 12.57 | 14.41 | 15.05 | 15.18 | 17.23 | 22.85 | 21.52 | 35.03 |
| Idaho Food Safety & Sanitation Manual | 12.16 | 12.87 | 13.43 | 15.25 | 16.02 | 16.61 | 20.63 | 20.77 | 26.39 |
| *FDA Food Code 2022* | 12.98 | 15.3 | 16.97 | 16.46 | 19.02 | 20.75 | 23.48 | 22.3 | 38.87 |

*Every headline quantity under each of the three extraction rule sets defined in Section 2.5.3. Permissive filtering discards only sentences above 400 tokens; strict filtering adds line-level removal of list, table and column-bleed content and tightens the sentence ceiling to 60 tokens; classification-based filtering applies the line-level removal and then drops individual sentences matching an audited artifact signature, with no length ceiling beyond a 400-token segmentation guard. FKGL denotes Flesch-Kincaid Grade Level, GFI the Gunning Fog Index, MSL mean sentence length in words. The federal Food Code is shown in italics and is never pooled with the guides.*

## Supplementary Table S2. Complete long-sentence audit (all sentences ≥ 80 tokens)

| **Document** | **Flagged** | **Artifact** | **Genuine** | **Artifact rate (%)** |
| --- | --- | --- | --- | --- |
| FDA Food Code 2022 | 306 | 114 | 192 | 37.3 |
| Idaho Food Safety & Sanitation Manual | 16 | 15 | 1 | 93.8 |
| Orange County (CA) Manual (ES) | 11 | 11 | 0 | 100.0 |
| Oregon Child Care Manual | 10 | 8 | 2 | 80.0 |
| Alaska Safe Food Worker Handbook | 8 | 2 | 6 | 25.0 |
| Orange County (CA) Manual | 8 | 5 | 3 | 62.5 |
| Southern Nevada HD Book (ES) | 8 | 8 | 0 | 100.0 |
| Oregon Child Care Manual (ES) | 8 | 8 | 0 | 100.0 |
| Oregon State Univ. Guide | 7 | 7 | 0 | 100.0 |
| Southern Nevada HD Book | 6 | 5 | 1 | 83.3 |
| Oregon Food Handler Manual | 5 | 3 | 2 | 60.0 |
| Oregon eFoodcard Study Guide | 4 | 1 | 3 | 25.0 |
| Washington Food Worker Manual | 4 | 1 | 3 | 25.0 |
| San Diego County (CA) Guide | 3 | 1 | 2 | 33.3 |
| San Diego County (CA) Guide (ES) | 3 | 3 | 0 | 100.0 |
| Lord Fairfax (VA) Manual | 3 | 2 | 1 | 66.7 |
| Washington Pacific Co. Booklet | 3 | 2 | 1 | 66.7 |
| Washington Food Worker Manual (ES) | 2 | 2 | 0 | 100.0 |
| Riverside County (CA) Guide | 1 | 1 | 0 | 100.0 |
| Rogers County (OK) Manual | 1 | 1 | 0 | 100.0 |
| **All documents** | **417** | **200** | **217** | **48.0** |

*Complete audit of every sentence in the corpus segmented at 80 tokens or more. This is a census rather than a sample: all 417 qualifying sentences were classified as genuine connected prose or as one of eight artifact types (contact directory, run-together bullet list, flattened numeric table, low alphabetic content, absence of function words, fragmented-token column bleed, heading run-on, absence of a finite verb). Classification features for every sentence are deposited. Note the divergence between tiers: long segments in the worker guides are predominantly artifacts, whereas the majority of those in the federal Food Code are authentic legal prose.*
